# Rapid manufacturing of angiogenic cellular collagen patches for ischemic cardiomyopathy

**DOI:** 10.1093/stcltm/szaf035

**Published:** 2025-09-19

**Authors:** Eric Pfrender, Sungwoo Kim, John A Farag, Shin Yajima, Yujiro Kawai, Koji Kawago, Umayr Syed, Gentaro Ikeda, Tsuyoshi Ueyama, Hiroyuki Takashima, Alex Dalal, Yuanjia Zhu, Kenzo Ichimura, Yu Liu, Seyedsina Moeinzadeh, Jayme Koltsov, Joseph C Wu, Y Joseph Woo, Phillip C Yang, Yunzhi P Yang, Yasuhiro Shudo

**Affiliations:** Department of Cardiothoracic Surgery, Stanford University School of Medicine, Stanford, CA 94305, United States; Stanford Cardiovascular Institute, Stanford University School of Medicine, Stanford, CA 94305, United States; Department of Orthopedic Surgery, Stanford University School of Medicine, Stanford, CA 94305, United States; Department of Cardiothoracic Surgery, Stanford University School of Medicine, Stanford, CA 94305, United States; Stanford Cardiovascular Institute, Stanford University School of Medicine, Stanford, CA 94305, United States; Department of Surgery, Stanford University School of Medicine, Stanford, CA 94305, United States; Department of Cardiothoracic Surgery, Stanford University School of Medicine, Stanford, CA 94305, United States; Stanford Cardiovascular Institute, Stanford University School of Medicine, Stanford, CA 94305, United States; Department of Cardiothoracic Surgery, Stanford University School of Medicine, Stanford, CA 94305, United States; Stanford Cardiovascular Institute, Stanford University School of Medicine, Stanford, CA 94305, United States; Department of Cardiothoracic Surgery, Stanford University School of Medicine, Stanford, CA 94305, United States; Stanford Cardiovascular Institute, Stanford University School of Medicine, Stanford, CA 94305, United States; Department of Cardiothoracic Surgery, Stanford University School of Medicine, Stanford, CA 94305, United States; Stanford Cardiovascular Institute, Stanford University School of Medicine, Stanford, CA 94305, United States; Stanford Cardiovascular Institute, Stanford University School of Medicine, Stanford, CA 94305, United States; Department of Medicine, Division of Cardiovascular Medicine, Stanford University School of Medicine, Stanford, CA 94305, United States; Department of Medicine, Division of Cardiovascular Medicine, Stanford University School of Medicine, Stanford, CA 94305, United States; Department of Medicine, Division of Cardiovascular Medicine, Stanford University School of Medicine, Stanford, CA 94305, United States; Department of Cardiothoracic Surgery, Stanford University School of Medicine, Stanford, CA 94305, United States; Stanford Cardiovascular Institute, Stanford University School of Medicine, Stanford, CA 94305, United States; Department of Cardiothoracic Surgery, Stanford University School of Medicine, Stanford, CA 94305, United States; Stanford Cardiovascular Institute, Stanford University School of Medicine, Stanford, CA 94305, United States; Stanford Cardiovascular Institute, Stanford University School of Medicine, Stanford, CA 94305, United States; Department of Medicine, Division of Pulmonary, Allergy, and Critical Care Medicine, Stanford University School of Medicine, Stanford, CA 94305, United States; Stanford Cardiovascular Institute, Stanford University School of Medicine, Stanford, CA 94305, United States; Department of Medicine, Division of Cardiovascular Medicine, Stanford University School of Medicine, Stanford, CA 94305, United States; Department of Orthopedic Surgery, Stanford University School of Medicine, Stanford, CA 94305, United States; Department of Orthopedic Surgery, Stanford University School of Medicine, Stanford, CA 94305, United States; Stanford Cardiovascular Institute, Stanford University School of Medicine, Stanford, CA 94305, United States; Department of Medicine, Division of Cardiovascular Medicine, Stanford University School of Medicine, Stanford, CA 94305, United States; Institute of Stem Cell Biology and Regenerative Medicine, Stanford University School of Medicine, Stanford, CA 94305, United States; Department of Cardiothoracic Surgery, Stanford University School of Medicine, Stanford, CA 94305, United States; Stanford Cardiovascular Institute, Stanford University School of Medicine, Stanford, CA 94305, United States; Department of Bioengineering, Stanford University School of Medicine, Stanford, CA 94305, United States; Stanford Cardiovascular Institute, Stanford University School of Medicine, Stanford, CA 94305, United States; Department of Medicine, Division of Cardiovascular Medicine, Stanford University School of Medicine, Stanford, CA 94305, United States; Department of Orthopedic Surgery, Stanford University School of Medicine, Stanford, CA 94305, United States; Department of Bioengineering, Stanford University School of Medicine, Stanford, CA 94305, United States; Department of Materials Science and Engineering, Stanford University School of Medicine, Stanford, CA 94305, United States; Department of Cardiothoracic Surgery, Stanford University School of Medicine, Stanford, CA 94305, United States; Stanford Cardiovascular Institute, Stanford University School of Medicine, Stanford, CA 94305, United States

**Keywords:** angiogenesis, biomaterials, collagen patch, endothelial progenitor cells, ischemic cardiomyopathy, myocardial infarction, tissue engineering

## Abstract

**Background:**

One in ten Americans carry a lifetime risk of ischemic heart failure, the most severe form of ischemic heart disease. Carrying a nearly 50% five‑year mortality rate, no interventional therapy exists to treat the underlying cause, microvascular malperfusion. In efforts to combat microvascular malperfusion, our group has utilized synergistic application of endothelial progenitor cells (EPCs) and smooth muscle cells (SMCs) to induce angiogenesis in ischemic myocardium.

**Methods:**

Cells are then embedded into a rapidly manufacturable compressed collagen (CC) patch to provide a biosimilar scaffold ready for transplantation. The performance of the cellular compressed collagen patch was then tested on a rodent acute myocardial infarction model of ischemic heart failure.

**Results:**

By post‑transplantation Day 28, the cellular CC patch improved left ventricular ejection fraction when compared to an acellular CC patch and control (cellular: 49.1 ± 1.8%; acellular: 38.0 ± 2.6%; control: 39.2 ± 2.1%; ANOVA *P* = .0006). Cellular CC patch transplantation also induced mature angiogenesis as shown by arteriolar density (cellular: 1084 ± 98 αSMA+vWF+/mm2; acellular: 338 ± 57 αSMA+vWF+/mm^2^; control: 449 ± 39 αSMA+vWF+/mm^2^; ANOVA P = .0003) and vascular maturation index (cellular: 0.67 ± 0.04; acellular: 0.48 ± 0.02; and control: 0.46 ± 0.04, *P* = .001).

**Conclusions:**

In conclusion, transplantation of a rapidly manufacturable EPC‑SMC‑based compressed collagen patch effectively rescues myocardial function by enhancing neovascularization and attenuating post‑infarction myocardial injury.

## Graphical abstract

**Figure:**
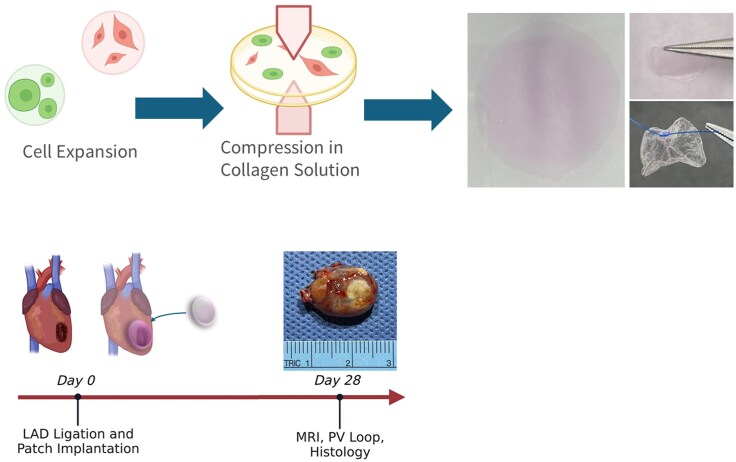

Significance statement Transplanting cellular compressed collagen patches prevents progression of ischemic heart failure by improving function and promoting angiogenesis.

We have successfully created a rapidly manufacturable compressed collagen patch with EPCs and SMCs to provide biosimilar scaffold ready for transplantation. The patch was transplanted into acute myocardial infarction model rats. The results demonstrated that the transplantation of this patch significantly improved myocardial function by promoting neovascularization and reducing post-infarction injury.

## Introduction

Ischemic heart disease remains a leading cause of death in the United States, with ischemic heart failure, its most severe form, on the rise. Americans face a 10% lifetime risk of developing ischemic heart failure, which has a staggering 46% five-year mortality rate.^1^^,^^2^ Driven by chronic coronary artery disease, acute myocardial infarction, and diabetes, ischemic heart failure currently lacks interventional therapy for two of its underlying causes. Both chronic coronary artery disease and diabetes are managed solely with medical optimization, as coronary intervention provides no clear benefit.^3^ While percutaneous coronary intervention and coronary artery bypass grafting are options for acute myocardial infarction, patients still face a 10% risk of developing ischemic heart failure within a year.^4^ This deterioration despite revascularization likely stems from untreated microvascular injury. The absence of effective therapies for microvascular damage leaves ischemic heart failure to progress unchecked, highlighting a critical gap in clinical care and an opportunity for innovative treatments to improve patient outcomes.

In an effort to treat ischemic heart failure, researchers have explored cell therapy,^4–7^ which targets mechanisms ranging from myocardial repair to angiogenesis. Several cell lines, including adult cardiac progenitor cells,^8^^,^^9^ induced pluripotent stem cells,^10^ endothelial progenitor cells,^11^ and mesenchymal stem cells (MSCs),^12^ have been tested individually or in combination.^13^^,^^14^ Angiogenic endothelial progenitor cells (EPCs) have shown promise for treating microvascular injury due to their high proliferative capacity and ability to form vascular networks in vivo.^15^ Mesenchymal stem cells also offer potential for cardiac disease treatment, given their multi-lineage differentiation, strong paracrine signaling, and homing abilities,^16^ and they have progressed to clinical trials for ischemic heart disease.^17^ However, EPCs alone have not fully realized their angiogenic potential due to inadequate support. Thus, we utilized the plasticity of MSCs to modulate them into a smooth muscle cell (SMC) phenotype, enhancing their paracrine potency and creating a supportive endothelial-mural cell relationship necessary for mature blood vessel formation.^18^

While the quest for viable therapeutic cell lines presented the initial significant challenge, the delivery platform has emerged as another obstacle. Initially focused on direct myocardial injection, cell therapies have evolved to encompass various delivery technologies aimed at addressing the limitations associated with intramyocardial or intracoronary cell injection,^19^ particularly regarding cell survival and retention. These strategies include tissue-engineered constructs such as cell sheets,^18^^,^^20^^,^^21^ hydrogel patches,^22^ and microencapsulation strategies,^9^ all designed to enhance cell retention at the site of injury. While previous tissue engineering approaches have improved the retention and effectiveness of transplanted cells, further optimization of current methodologies is necessary to enhance efficiency and reproducibility. Moreover, the integration of diverse cell types into engineered tissues remains complex and time-intensive, prompting the exploration of innovative strategies for cell delivery and organization.^5^

In this study, collagen-based biomaterials were employed to deliver EPCs and SMCs in a myocardial infarction model. Collagen, a pivotal constituent of the extracellular matrix (ECM), assumes a crucial role in tissue engineering and regenerative medicine. Its exceptional biocompatibility fosters favorable conditions for cell attachment and proliferation, rendering it an ideal choice for applications in tissue regeneration.^23–25^ Integrins facilitate cell adhesion to collagen, thereby promoting conducive conditions for cell growth, differentiation, and migration.^26^ Nonetheless, the extensive utilization of collagen-based biomaterials for tissue engineering scaffolds is frequently hindered by their insufficient mechanical and physical properties.^23^^,^^27^^,^^28^ Consequently, numerous studies have sought to enhance the mechanical, physical, and biological properties of collagen-based biomaterials by optimizing of their macro- and microstructures using crosslinking and fabrication methods.^23^^,^^27^^,^^29^

In this context, plastic compression (PC) of collagen hydrogel has emerged as a promising physical method for reinforcing hydrogel mechanical integrity without causing toxicity.^30–32^ By incorporating cells into the hydrogel before compression, their viability post-compression is ensured, while the interconnected collagen fibrils maintain their integrity throughout the process, bolstering the resulting material.^26^^,^^28^^,^^30^^,^^31^^,^^33^ The compressed collagen patch offers significant advantages over collagen hydrogel, addressing challenges such as poor tensile properties and suturing difficulties. This improvement facilitates more accurate replication of the native matrix in clinical applications.^28^ The compressed collagen patch facilitates ease of handling during surgery, combining flexibility and strength without the risk of rupture. This capability to generate matrices with tissue-like mechanical strength enables direct transplantation, affording precise control over dimensions and mechanical properties.^26^^,^^28^^,^^34^ The compression process yields a collagen sheet consisting of compacted layers of fibrils, with the highest density near the fluid-leaving surface.

Given the proven efficacy of synergistic EPC-SMC angiogenesis and myocardial protection,^18^^,^^21^ we have integrated these delicate cells with a durable collagen-based biomaterial and applied it in the setting of acute MI. Our transplantation-ready cellular compressed collagen (CC) patches can be assembled in less than an hour and are fabricated by integrating EPCs and SMCs into a collagen type I solution, allowing for hydrogel formation, and then subjecting it to PC. These cellular CC patches maintain cell viability and approach tissue-like cell density with high mechanical robustness and flexibility that allows the patch to be easily handled during surgery. Therefore, we hypothesize that a rapidly manufacturable EPC-SMC-based compressed collagen patch will rescue myocardial function by enhancing neovascularization and attenuating post-infarction myocardial injury. We hypothesize that functional recovery is realized through both “direct” cell incorporation into the host myocardium leading to angiogenesis and “indirect” paracrine signaling that bolsters auto-angiogenesis.

## Methods

### EPC isolation and culture

Human EPCs were isolated from healthy female donor peripheral blood (STEMCELL Technologies, Vancouver, Canada), as previously described.^13^^,^^18^^,^^21^^,^^35^ Mononuclear cells were collected via density-gradient centrifugation: Blood was mixed with phosphate buffered saline (PBS), layered over 15 ml Lymphoprep (STEMCELL Technologies, Vancouver, Canada), and centrifuged for 30 minutes at 560 × g. The mononuclear cells in the buffy coat layer were carefully collected and washed in PBS by centrifuging at 300 × g for 10 minutes at room temperature (RT) followed by aspiration and resuspension. After 3 washes, EPCs were resuspended in EGM-2 BulletKit (Cat. CC-3162; Lonza Inc., NJ, United States). Isolated EPCs were seeded in 6-well plates coated with fibronectin at a density of 50 × 10^6^ cells/well and cultured in a humidified incubator at 37 °C with 5% CO_2_. Media was exchanged every 24 hours for the first seven days to remove non-adherent cells, followed by every 48 hours thereafter. Colonies exhibiting the EPC cobblestone morphology began to appear at day 10-14, with confluency achieved at day 24-28. After the first passage, cells were cultured on 0.2% gelatin substrate. Cells passage 4-9 were used for assays and in vivo transplantation.

To characterize isolated EPCs, immunocytochemistry for CD31, CD34, vWF and VEGFR-2 was performed on cultured cells at day 3.^16^ Cells were cultured in 96-well plate until confluent, washed 3× with PBS, and fixed with 4% paraformaldehyde. After washing again with PBS, cells were permeabilized with 0.5% TWEEN-20/PBS, washed, and blocked with 10% goat serum for one hour. Primary antibodies were added and incubated overnight at 4C. The next day, cells were incubated with secondary antibodies for 1 hour at RT and imaged with a BZ-X800 fluorescence microscope (Keyence, Osaka, Japan).

### MSC modulation to SMC-like cells and culture

SMC-like cells were modulated from MSCs, as previously described.^13^^,^^18^^,^^21^^,^^36^ Human bone marrow-derived MSCs were acquired from a commercial source (Lonza, Inc., NJ, United States) and cultured in minimum essential medium alpha (MEMa, Life Technologies, CA) supplemented with 10% fetal bovine serum (FBS), 1% GlutaMAX (Gibco, Life Technologies, CA), and gentamicin at 37 °C with 5% CO_2_. Cells were plated at 4-6 × 10^6^ cells/cm^2^, and medium was exchanged every 3-4 days. For modulation to the SMC phenotype, cells at approximately 85% confluency were lifted and plated at a density of 4-6 × 10^6^ cells/cm^2^ in dishes coated with human plasma fibronectin in Smooth Muscle Growth Medium-2 BulletKit (SmGM-2, Lonza, Inc., NJ) supplemented with 2 ng/µl TGF-beta (Sigma, Aldrich, St Louis, MO).^36^ Cells (referred to as SMCs throughout the manuscript) were expanded for several passages in the SMC medium and fibronectin coating before use in biological assays and in vivo transplantation. SMCs were used at passage 5-10.

To confirm SMC phenotype, SMC-like MSCs were characterized using immunocytochemical staining for alpha smooth muscle actin (αSMA), SM22α, and caldesmon at day 3.^37^ Cells were cultured in 96-well plate until confluent, washed 3× with PBS, and fixed with 4% paraformaldehyde. After washing again with PBS, cells were permeabilized with 0.5% TWEEN-20/PBS, washed, and blocked with 10% goat serum for 1 hour. Primary antibodies were added and incubated overnight at 4 °C. The next day, cells were incubated with secondary antibodies for 1 hour at RT and imaged with a BZ-X800 fluorescence microscope.

### Compressed collagen patch formation

The CC patch was engineered to impart tissue-like physical and biological attributes, such as flexibility, mechanical strength, and biocompatibility. This was achieved through a compressive dehydration and post-processing approach.^28^^,^^31^ Modulating the physical and mechanical properties of the CC patch was straightforward, as it involved adjusting parameters such as compression duration, load, volume, and collagen solution concentration. For this study, we prepared cellular and acellular CC patches separately. A mixture of 1.5 × 10^6^ EPCs and 1.5 × 10^6^ SMCs was combined with 500 µl of collagen solution (3 mg/ml, type I, rat tail), transferred into a sterile casting reservoir (a cylindrical Teflon mold with 10 mm diameter and 8 mm thickness), and then incubated at 37 °C for 50 minutes to form a collagen hydrogel. Subsequently, the cell-laden collagen hydrogel was placed between sterile absorbent filter papers and compressed using a mass of 18 g for 5 minutes to expel fluid rapidly. Following compression, the cellular CC patches were deemed transplantation-ready and stored in cell culture media at 37 °C until transplantation. Acellular CC patches were also prepared using the same protocol without addition of cells.

### Mechanical testing

Mechanical properties of collagen hydrogels and CC patches were assessed through tensile testing. The collagen hydrogel was subjected to compression using an 18 g mass for durations of 0, 2, and 5 minutes to evaluate the effects of compression duration. Cross-sectional area was determined from three locations along the specimen gauge length. Tensile testing was conducted using a Model 5944 test system (Instron Corp., Norwood, MA) equipped with a 10 N load cell (Interface, Scottsdale, AZ) and a saline bath chamber to maintain sample hydration. Samples were preloaded with approximately 5 mN and subjected to uniaxial loading at a rate of 1% strain per second until failure. Tensile modulus was calculated as the initial linear slope of the stress-strain curve from 0% to 1% strain. All tests were performed in PBS solution at 37 °C.

### Cell proliferation in vitro assessment

Human MSCs (hMSCs) were cultured in Dulbecco’s modified eagle medium (Gibco, Life Technologies, CA) supplemented with 10% FBS, 1% antibiotic/antimycotic mixture, 5 ml of L-glutamine (200 mM), and sodium pyruvate at 37 °C in a 5% CO2 humidified incubator. The cells were encapsulated into the CC patch at a density of 1 × 10^6^ cells/sample and cultured in 12-well plates for 4 weeks. Cell viability and proliferation rate were quantitatively assessed at day 0, 1, 3, 7, 14, 21, and 28 using an MTS assay following the manufacturer’s instructions. The optical density at 490 nm was measured using a microplate reader (TECAN Infinite F50, Männedorf, Switzerland).

### Myocardial infarction model and CC patch transplantation

All animals were handled according to protocols approved by the Institutional Animal Care and Use Committee (APLAC protocol #34273). Male T-cell-deficient, athymic nude rats (RNU, 8 weeks old, 200-250 g) were obtained from Charles River (Wilmington, MA, United States).

Procedures were performed under general anesthesia. The AMI model was created by permanent ligation of the left anterior descending artery (LAD). The LAD was permanently ligated with a 6-0 monofilament suture in a proximal location (2 mm below the left atrial appendage) via a left thoracotomy. Infarct size was confirmed to encompass 40% of left ventricle (LV) wall by LV pallor. Rats were allocated to each experimental group: control (MI-only) (n = 18), acellular CC patch (n = 14), or cellular CC patch (n = 21). Additionally, *n* = 8 rats received cellular CC patches with lentivirus-transduced EPCs and SMCs after LAD ligation which were not included in functional analysis experiments, and *n* = 3 rats received a cellular CC patch after thoracotomy with no LAD ligation (Sham). CC patches were placed on the infarct area below the ligation suture and used the same suture to secure the patch. Fibrin tissue glue (TISSEEL, Baxter International, Deerfield, IL) was placed around the edge of the patches to fix them to heart’s surface.

### Cardiac MRI and MEMRI for myocardial functional and viability assessment

Cardiac functional assessment was performed by magnetic resonance imaging (MRI) in rats post-MI Day 1 and again at post-MI Day 28 (cellular CC patch *n* = 17, acellular CC patch *n* = 10, and control *n* = 14). Cardiac MRI (CMR) was performed on a Signa 3 T EXCITE scanner (GE Healthcare, WI, United States) and a phased array 4 channel surface coil (Rapid MR international).^38^ Short-axis serial slices were obtained to determine LV ejection fraction, LV end-systolic volume (LVESV), LV end-diastolic volume (LVEDV), and LV mass. Manganese-enhanced MRI (MEMRI) was used to differentiate viable and non-viable myocardium; images were collected using an IR_FSPGR sequence 60 minutes following EVP1001-1 administration (8µL/g, IP, Eagle Vision Pharmaceutical Corp., Downington, PA).^39^ Rats with LV ejection fraction greater than 50% at post-MI Day 1 were determined to not have a significant infarction, and therefore excluded from all further analyses.

### Invasive hemodynamic analysis and heart explant

Four weeks following myocardial infarction procedure, animals underwent invasive hemodynamic analysis prior to sacrifice (cellular CC patch *n* = 10, acellular CC patch *n* = 5, and control *n* = 9). Under general anesthesia, median sternotomy was performed, and the pressure volume conductance catheter (SPR-869; Millar Instruments, Inc, Houston, TX) was inserted into the left ventricle through the LV apex.^40^ Pressure volume loops were recorded before and during occlusion of the inferior vena cava. Data recording and analysis were conducted in LabChart and Millar software (ADInstruments, Dunedin, New Zealand). Volume calibration was achieved using LV volume measurements from MRI data. One additional rat in the cellular CC patch was excluded for an extreme outlier pressure reading.

Following PV Loop data acquisition, hearts were explanted for RNA sequencing and histology. For RNA sequencing, samples were taken from the infarction area and remote area (septal tissue) by visual examination. A separate group of animals (cellular CC patch *n* = 4, acellular CC patch *n* = 4, and control *n* = 4) were sacrificed at post-MI Day 7 following ligation, and samples were saved for RNA sequencing.

### Histological and immunohistochemical analysis

Heart tissues were stained with hematoxylin-eosin (­Rapid-Chrome H&E Frozen Section Staining Kit, Epredia, Kalamazoo, MI) and Masson’s Trichrome (Masson’s Trichrome Stain Kit, StatLab, McKinney, TX). Slides were imaged using Keyence BZ-X800 microscope; for fibrosis analysis, fibrotic area was calculated as the percentage of the left ventricle stained blue by Masson’s Trichrome. We distinguished the left ventricles to three sections: infarct, unaffected(remote), and infarct border zone. Three sections were taken from each heart, and 5 images were randomly captured around the infarct and border zone on each section. For each heart, 4 distinct sections were evaluated and averaged. For immunohistochemical (IHC) staining, anti-αSMA (1:100, Abcam, Cambridge, UK, Cat: ab21027), anti-von Willebrand Factor (vWF) (1:100, Abcam, Cambridge, UK, Cat: ab6994), anti-Cardiac Troponin T (1:100, Abcam, Cambridge, Cat; ab8295), and anti-Collagen type I (1:100, Sigma-Aldrich, Saint Louis, United States, Cat: C2456) antibodies were used as primary antibody. Frozen slides were thawed briefly and washed 3× in TBS + 0.025% Triton-X 100, followed by fixation for 10 minutes in 4% paraformaldehyde, permeabilization for 15 minutes in PBS + 0.5% TWEEN, and blocking using 10% donkey serum for 1 hour. The slides were then incubated overnight at 4 °C with primary antibodies diluted in TBS + 1% BSA. The next day, slides were washed 3× and incubated with secondary antibodies diluted in TBS + 1% BSA for 1 hour at RT. Following a final 3× wash, slides were coated with VECTASHIELD mounting medium with DAPI (Vector Laboratories, Newark, CA) and a coverslip, and stored in the dark at 4 °C until they were imaged using the Keyence microscope. Microvascular density was calculated using Keyence analysis software to identify the number of αSMA+vWF+ arterioles per 20x image field. The images were taken randomly from the border zone area, with 15 images used for each rat. We additionally calculated the vascular maturation index (VMI), percentage of mature arterioles of all arterioles, by dividing αSMA+vWF+ cells αSMA−vWF+.^41^^,^^42^

### Bulk RNA sequencing and pathway enrichment analysis

RNA was isolated from flash frozen tissues of infarct area and border zone area post-MI Day 7 (cellular CC patch *n* = 4, acellular CC patch *n* = 4, control *n* = 4), using the Qiagen miRNeasy Isolation Kit (Qiagen, Germantown), followed by cleaning with RNA Clean and Concentrator Kit (Zymo Research, Irvine, CA).

For RNA-seq data, quality was examined by way of analyzing per base sequence quality plots using FastQC. The trimming of sequence reads was done by TrimGalore. RNA-seq reads were aligned to the rattus norvegicus genome (mRatBN7.2) using the STAR software.^43^ Reads that overlapped with exon coordinates were counted using featurecounts.^44^ Raw read counts were transformed using the variance stabilizing transformation (VST) function included in the DESeq2 R package. Mean and SD of normalized expressions were calculated for each gene. Z-scores were determined by subtracting the mean from each expression value and dividing by the SD. Differentially expressed genes (DEGs) between different groups were identified using DESeq2 R package.^45^ Unsupervised differential expression with default parameters was evaluated between infarct samples between each group (for example, CC patch group infarct vs control group infarct). Genes with a Benjamin-Hochberg corrected *P* < .05 were considered significant. The functions and pathways of the DEGs were predicted using the gene ontology annotation (GO), Kyoto Encyclopedia of Genes, and Genomes (KEGG) and Reactome database by the web server KOBAS.^46^ FDR < 0.05 was set as a threshold of significance.

### Lentiviral transduction and bioluminescent in vivo imaging

EPCs and SMCs were stably transduced with a double reporter construct for the dual expression of luciferase and turbo Green Fluorescent Protein (turboGFP) for EPCs or turbo Red Fluorescent Protein (turboRFP) for SMCs under a cytomegalovirus promoter to drive ubiquitous expression.^47^ Cells were plated in a six-well plate at a density of 4 × 10^6^ cells/cm^2^. Transduction-ready third generation lentiviral particles (Cat: LV-1016, LV-1017, FenicsBIO, Halethrope) were introduced to the culture medium at an approximate multiplicity of infection of 5 along with 8 μg/mL polybrene (Gibco, Life Technologies, CA). Plates were centrifuged at 1000 × g for 1 hour at 32 °C to maximize viral particle contact with the cells. Culture medium was exchanged after 24 hours. At 48 hours post-transduction, 0.5 µg/ml puromycin was used to select for successfully transduced cells and exchanged every other day for 4 days.

Using the stably transduced EPC and SMC cell lines, compressed collagen patches were created and transplanted into animals immediately following LAD ligation. A subset of animals (n = 3) received cellular CC patch transplantation with no LAD ligation. Following transplantation, bioluminescent images were taken of the animals at post-transplantation Hour 4, Day 1, Day 3, Day 7, Day 10, Day 14, Day 21, and Day 28. Under general anesthesia, D-Luciferin (150 mg/kg IP, GoldBio, St Louis, MO) was administered, and images were taken every 10 minutes on an SII Lago-X (Spectral Instruments Imaging, Tucson, AZ) until peak emission strength was reached (approximately 40-60 minutes after D-Luciferin injection). Animals were sacrificed on post-transplantation Day 1 (n = 2), Day 7 (n = 2), and Day 28 (n = 7, including *n* = 3 with no MI), and hearts were frozen for histology as previously described. For visualization of transplanted cells, 10-µm-thick cryosections were stained with anti-turboGFP (1:300, ThermoFisher, United States, Cat: TA150071) and anti-turboRFP primary antibodies (1:100, ThermoFisher, United States, Cat: TA150061) followed by incubation with appropriate secondary antibodies (1:200, Abcam, Cambridge, UK). The slides were also stained with anti-von Willebrand Factor (vWF) (1:100, Abcam, Cambridge, UK, Cat: ab6994).

### Statistics

Continuous variables were presented as means and SEs. Normality was assessed using Shapiro-Wilk tests. When normally distributed, groups were compared with single-factor ANOVAs, followed by post hoc Tukey-Kramer tests. Analyses were performed via JMP, version 16.2 (SAS Institute, Inc., Cary, NC) and Prism version 9.5.1 (GraphPad Software, La Jolla, CA), with a two-sided level of significance of α = 0.05

## Results

### Characterization of human cells and CC patches

**Figure 1.**
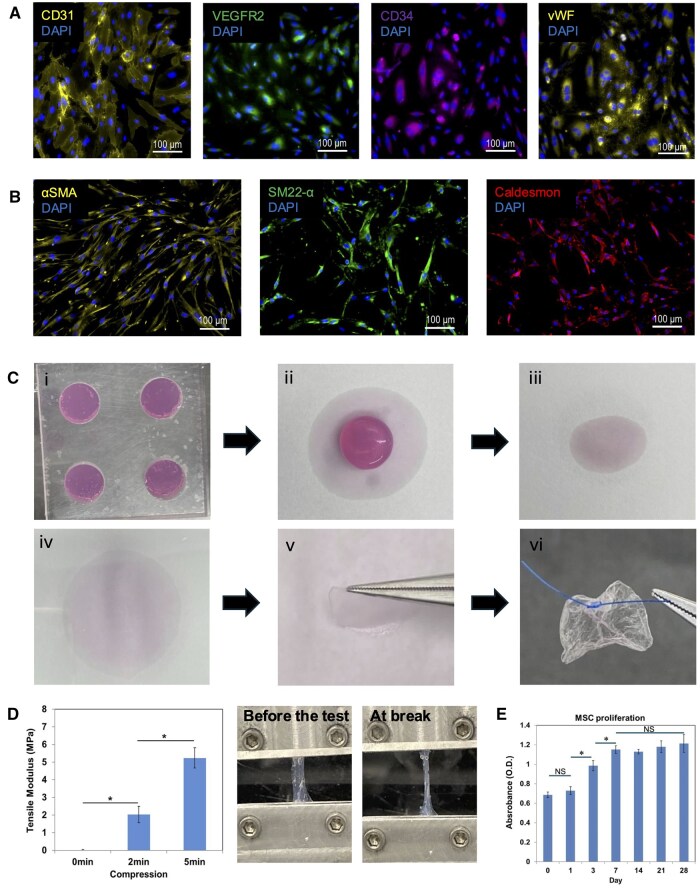
In vitro characterization of isolated EPCs and SMCs and cellular CC patch. (**A**) Immunostained EPCs for markers CD31, VEGFR2, CD34 and vWF. DAPI is used for nuclear stain; scale bar is 100 mm. (**B**) Immunostained SMCs for markers αSMA, SM22α and Caldesmon. DAPI is used for nuclear stain; scale bar is 100 mm. (**C**) Creation of CC patch **i**. hydrogel formation in a Teflon mold at 37 °C for 50 minutes, **ii**. Hydrogel placed in sterile absorbent filter paper, **iii**. Additional paper placed atop hydrogel, **iv**. Hydrogel compressed using 18 g mass for 5 minutes to expel fluid rapidly, **v**. a flexible, durable CC patch is ready for implantation, **vi**. Image demonstrating suturability. (**D**) tensile modulus of the CC patch according to compression duration and images demonstrating elasticity (n = 3). (**E)** SMC-like MSCs proliferation in the CC patch during 4 weeks of cell culture (n = 4). Error bars represent ± SD; (NS) indicates no significance, (*) Indicates *P* < .05. EPC indicated endothelial progenitor cell; SMC—smooth muscle cell; MSC—mesenchymal stem cell. (ANOVAs, post hoc Tukey-Kramer tests, *P* < .05).

Characterization of isolated human cells and the CC patches were conducted in vitro. Human peripheral blood-derived EPCs were positive by immunostaining for CD31, CD34, VEGFR2, and vWF (Fig. 1A). Human bone marrow-derived MSCs were directed toward a SMC lineage by supplementation with Smooth Muscle Growth Medium-2 and TGF-b. Immunostaining of SMC-like MSCs, henceforth referred to as SMCs, showed expression of SMC markers αSMA, SM22α, and Caldesmon (Fig. 1B). Cultured EPCs and SMCs were encapsulated in collagen type 1 solution at a concentration of 1.5e6 EPCs + 1.5e6 SMCs per patch; 3 million total cells were found to be the upper limit for reliable patch formation. Following gelation, PC enhanced the stability and mechanical strength of the patch, resulting in increased flexibility and suturability, thereby facilitating ease of transplantation (Fig. 1C).

The tensile modulus of the CC patch significantly increased with compression duration (0 min compression: 0.015 MPa, 2-min compression: 2.03 MPa, 5-min compression: 5.23 MPa). This resulted in approximately 135-fold and 349-fold increases in the tensile modulus of the CC patch with 2- and 5-minutes compression, respectively, compared to collagen hydrogel (*P* < .05) (Fig. 1D).

After 3 days of culture, metabolic activity of SMC-like MSCs increased (*P* < .05). Cells exhibited notably higher metabolic activity and achieved a high level of confluency at day 3 compared to day 0 and 1 (*P* < .05), suggesting the non-cytotoxic nature of the collagen membrane (Fig. 1E). Cell proliferation was significant, reaching a plateau by day 7, and maintaining confluency within the collagen patch for the entire 4-week period. No significant differences were observed among different time points after 7 days of cell culture. These results highlight the cell-friendly nature of the compressive dehydration method as a casting process and indicate that the compressed collagen patch effectively supports cell growth and proliferation.

### Cardiac functional recovery following CC patch transplantation

**Figure 2.**
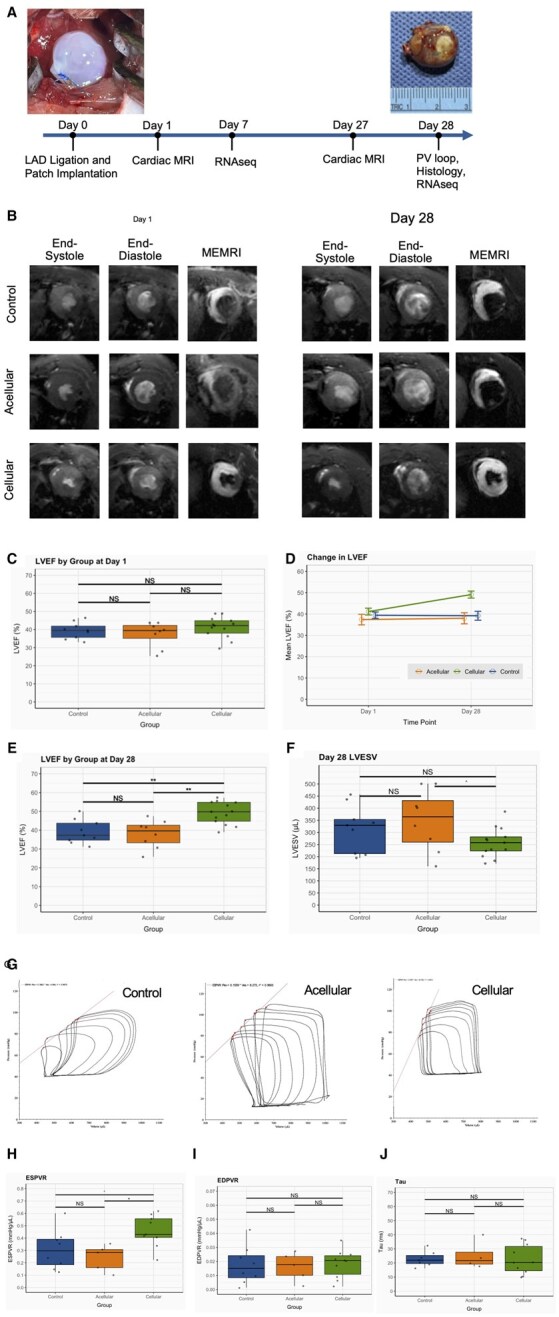
MRI and PV-loop assessment of cellular CC patch. **(A)** Study timeline showing Day 0 cellular CC patch implantation on the infarcted left ventricle and Day 28 cellular CC patch on the explanted heart. The control group (n = 14), acellular CC patch group (n = 10), and cellular CC patch group (n = 17) were included in the MRI study, while PV-loop assessment was performed in the control group (n = 9), acellular group (n = 5), and cellular group (n = 10). (**B)** Representative images of cardiac MRI showing end-systole, end-diastole, and MEMRI for each group at Days 1 and 28. (**C)** Left ventricle ejection fraction (LVEF) MRI data at post-MI Day 1. (**D)** LVEF change (mean ± SE) from Day 1 to Day 28. (**E)** LVEF MRI data at post-MI Day 28. (**F)**, LVESV on post-MI Day 28. (**G)** Representative image of control group PV loop, acellular CC patch group PV loop, and cellular CC patch group PV loop. (**H)** End-systolic pressure-volume relationship (ESPVR) at Day 28. (**I)** End-diastolic pressure-volume relationship (EDPVR) at Day 28. **(J)** Tau at Day 28. Box plots show medians (central line), interquartile range (edges of box) and 1.5× interquartile range (whiskers). (NS) indicates no significance, (^) indicates *P* < .1, (*) indicates *P* < .05, (**) indicates *P* < .01. LAD—left anterior descending artery; LVEF—left ventricle ejection fraction; ESPVR—end-systolic pressure-volume relationship. (ANOVAs and Tukey-Kramer tests, *P* < .05).

The effects of the cellular CC patch on cardiac function and remodeling were evaluated using CMR and pressure-volume conductance catheters (Fig. 2A). Representative CMR images of end-systole, end-diastole, and MEMRI from post-MI Day 1 and Day 28 are shown for each group (Fig. 2B). There was no difference in the viable myocardial area as designated by MEMRI in any group at Day 1 (cellular: 76.5 ± 2.62%; acellular: 76.4 ± 4.25%; control: 73.9 ± 2.78%; ANOVA *P* = .8) or day 28 (cellular: 85.0 ± 1.8%; acellular: 82.3 ± 2.7%; control: 83.8 ± 1.7%; ANOVA *P* = .7). The change in viable myocardial area increased in each group; however, there was no difference between groups (ANOVA *P* = .6) (Table S1). On post-MI Day 1, LVEF (Fig. 2C), LVESV, and LVEDV showed no difference between groups (Table S1). However, in the cellular CC patch group LVEF increased (+7.96 ± 0.87%, *P* = .019) over time, whereas the other groups did not change (Fig. 2D). At Day 28, LVEF was highest in the cellular CC patch group (cellular: 49.1 ± 1.8%, acellular: 38.0 ± 2.6%, control: 39.2 ± 2.1%, ANOVA *P* = .0006, Fig. 2E). The cellular CC patch group also had tendency of a lower LVESV at Day 28 (cellular: 258 ± 32µL; acellular: 348 ± 32µL; control: 316 ± 31µL; ANOVA *P* = .09, Fig. 2F), but did not achieve significance.

Invasive hemodynamic analysis was performed at post-MI Day 28 to elaborate on evidence of functional recovery found in the MRI data. Representative Pressure-Volume (PV) Loops from post-MI Day 28 are shown for each group (Fig. 2G). At Day 28, the cellular group showed higher end-systolic pressure-volume relationship (ESPVR) than the other groups (cellular: 0.456 ± 0.043; acellular: 0.241 ± 0.048; control: 0.307 ± 0.057 mmHg/µL, ANOVA *P* = .02, Fig. 2H), demonstrating improved intrinsic LV myocardial contractility. Importantly, the epicardial fixation of the CC patch did not cause diastolic dysfunction as end-diastolic pressure-volume relationship (EDPVR, ANOVA *P* = .9) and Tau (ANOVA *P* = .9) were not different between groups (Fig. 2I-J).

### Reduced fibrosis and enhanced microvascular density

**Figure 3.**
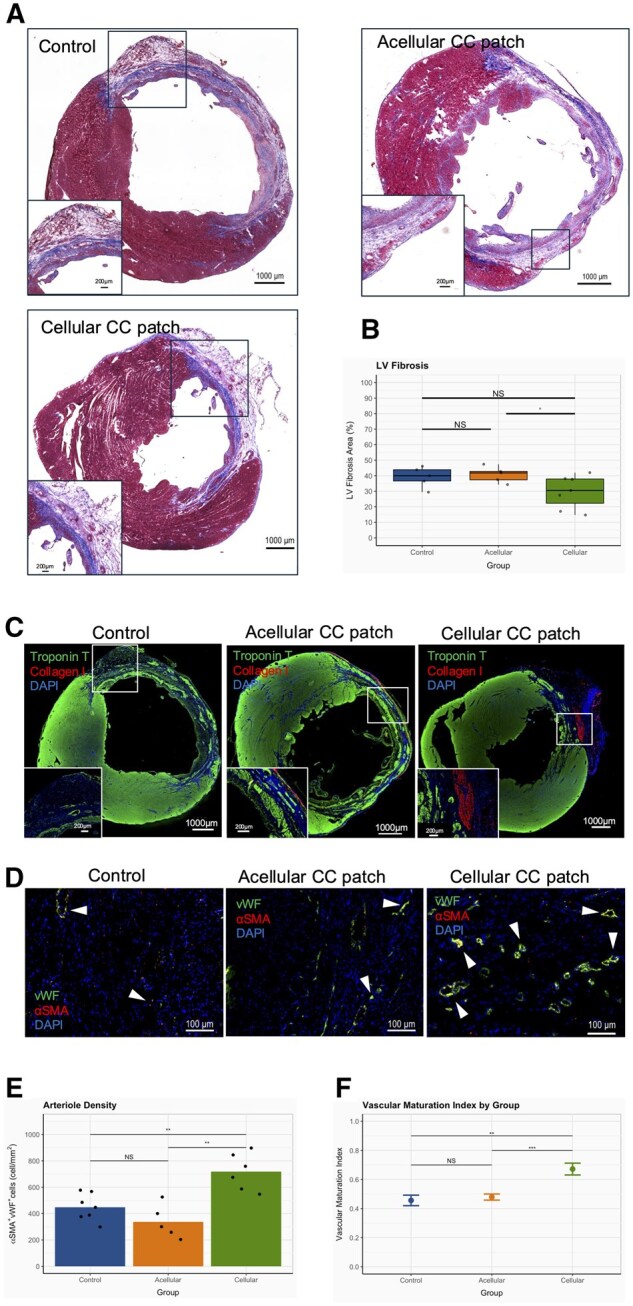
Histological examination at 4 weeks after MI. (**A**) Masson’s Trichrome staining of hearts shows viable myocardium and fibrotic scar. Inserted boxes present high magnification. **(B)** Comparison of LV fibrosis area between groups estimated from Masson’s Trichrome staining. Control(n = 7), Acellular(n = 5), Cellular CC patch(n = 7). (**C**) Immunostaining for cardiac troponin T , collagen type 1 , and DAPI nuclear stain. Inserted boxes present high magnification. (**D)** Immunostaining for microvascular density; endothelial marker (vWF), smooth muscle marker (αSMA), and DAPI nuclear stain. Arrowheads indicate double-positive cells. **(E**) Bar graph and accompanying data points indicating arteriole density (αSMA+vWF+ cells/mm^2^) for each group. Control (n = 7), acellular (n = 5), cellular CC patch (n = 6). **(F)** Vascular maturation index (αSMA+vWF+ cells/vWF+ cells) shown for each group presented as (mean ± SE). (^) Indicates *P* < .1, (*) indicates *P* < .05, (**) indicates *P* < .01. LV, Left ventricle; vWF, von Willebrand Factor; αSMA, alpha Smooth muscle actin. (ANOVAs and Tukey-Kramer tests, *P* < .05).

Histological analysis of the fibrotic scar by Masson’s Trichrome staining revealed LV anterior walls exhibit thinning and collagenous scar formation consistent with severe MI at Day 28: (Fig. 3A). Some cellular CC patch hearts show reduced infarct area, while hearts that received acellular CC patches tended to have an increased area compared to both cellular and control groups (Fig. 3B). Immunostaining for cardiac troponin T and collagen I revealed the extent of troponin T loss in the infarct area and the presence of residual collagen on the surface in all groups. (Fig. 3C). Residual collagen was observed on the surface of the scar area in both the acellular CC patch and cellular CC patch groups, while cardiomyocyte loss (indicated by the area of negative cardiac troponin T staining) was reduced in cellular CC patch treated hearts. Staining for endothelial (vWF) and SMC markers (αSMA) in border zones showed αSMA+vWF+ cell clusters, representing mature arterioles, in each group (Fig. 3D). The cellular CC patch group also shows an increased arteriolar density (cellular: 1084 ± 98 αSMA+vWF+/mm^2^; acellular: 338 ± 57 αSMA+vWF+/mm^2^; control: 449 ± 39 αSMA+vWF+/mm^2^; ANOVA *P* = .0003) (Fig.3E). Moreover, a high number of these arterioles are mature vessels as indicated by VMI. Again, the cellular CC patch group VMI was highest amongst groups (cellular: 0.67 ± 0.04; acellular: 0.48 ± 0.02; and control: 0.46 ± 0.04, *P* = .001) (Fig. 3F).

### Transcriptomics analysis

**Figure 4.**
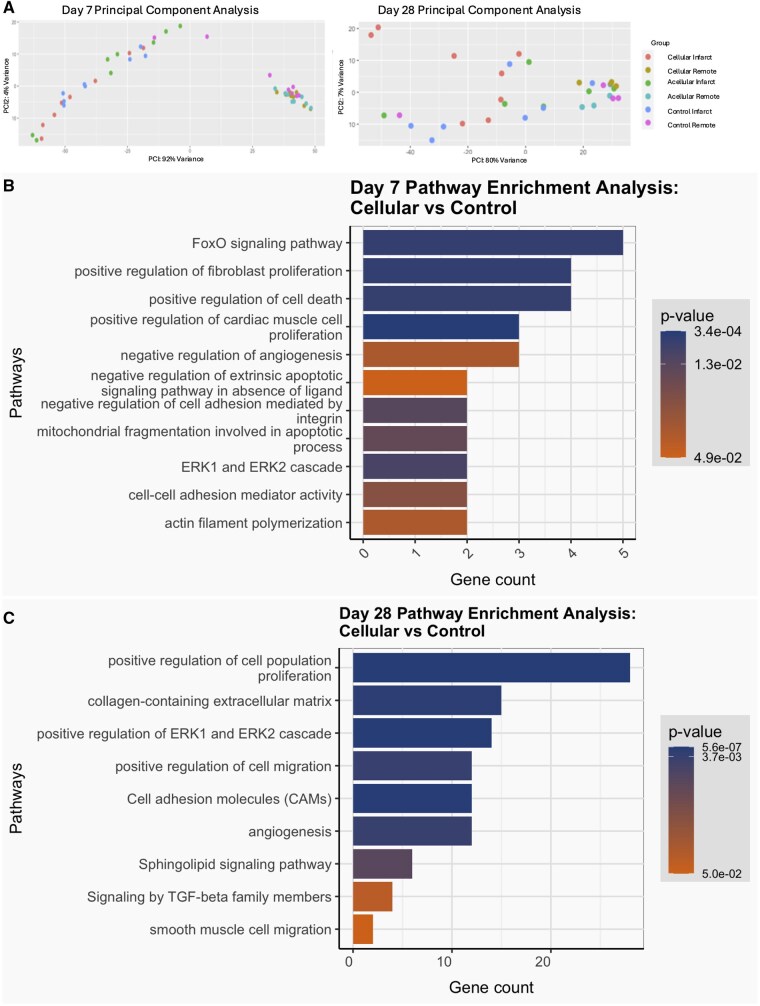
RNA sequencing analysis of the myocardial samples form infarct area and border zone area at post-MI Day 7 and Day 28. Control (n = 4), acellular (n = 4), cellular CC patch (n = 4). (**A**) Principal component analysis of samples at post-MI Day 7 and post-MI Day 28. **(B)** Pathway enrichment analysis (PEA) of cellular CC patch vs Control group at post-MI Day 7 showing relevant enriched pathways. **(C)** PEA of Cellular CC patch vs control group at post-MI Day 28 showing relevant enriched pathways. Bar plots show Gene Ontology Pathway names with gene counts and *P* values.

To further understand the molecular mechanisms by which the cellular CC patch rescues the ischemic myocardium, we performed bulk RNA sequencing on the three experimental groups using tissue samples taken at post-MI Days 7 and 28 days. Principal component analysis revealed that samples from remote locations within each group tended to cluster together and samples from the infarct area formed a distinct and separate cluster, thus revealing underlying patterns and variations in gene expression profiles (Fig. 4A). At post-MI Day 7, the cellular CC patch group infarct area exhibited 338 upregulated and 131 downregulated genes compared to the control group, and 124 upregulated and 52 downregulated genes compared to the acellular CC patch group. At post-MI Day 28, there were 616 upregulated and 430 downregulated genes compared to control, and 139 upregulated and 36 downregulated genes compared to the acellular CC patch group.

We then performed pathway enrichment analysis to investigate differences between groups. When compared to the control group at Day 7, the cellular CC patch group exhibited increased angiogenesis-related pathways as evidenced upregulation of the ERK1 and ERK2 cascade (endothelial cell proliferation)^48^ and downregulation of negative regulation of angiogenesis.^49–51^ Additionally, the cellular CC patch group exhibited increased cardiomyocyte survival as evidenced by upregulation of cardiac muscle cell proliferation^52^ and FoxO signaling pathway (enhanced antioxidant defense)^53^ along with downregulation of cell death and mitochondrial fragmentation involved in the apoptotic process. The cellular CC patch group had increased cellular adhesion and decreased anoikis^54^ as evidenced by upregulation of cell-cell adhesion mediator activity and actin filament polymerization and downregulation of negative regulation of cell adhesion mediated by integrin and negative regulation of extrinsic apoptotic signaling pathway in the absence of ligand. There is also evidence of improved extracellular remodeling as shown by upregulation of fibroblast proliferation (Fig. 4C).

By Day 28, the cellular CC patch again showed more evidence of robust angiogenesis pathways with upregulation of angiogenesis^55–58^ and ERK1 and ERK2 cascade and downregulation of sphingolipid signaling pathway (reduces endothelial cell apoptosis).^59^ There were continued signs of cell adhesion and migration as shown by upregulation of cell adhesion molecules,^56^^,^^60^ positive regulation of cell migration (adult stem cells and macrophages),^61^^,^^62^ and SMC migration. Additionally, there was evidence of positive extracellular matrix remodeling as there was upregulation of pathways related to collagen-containing extracellular matrix. This pathway includes genes that are critical for fibrosis prevention,^63–65^ tissue elasticity maintenance,^66^ and tissue growth and repair.^58^^,^^67^ There was also downregulation of TGF-beta family members, which is also critical in preventing fibrosis (Fig. 4D).

When comparing the cellular CC patch and the acellular CC patch groups at Day 7, the key difference is a gene involved positive regulation of endothelial cell chemotaxis, response to fluid shear stress, and cardiac muscle contraction (P2rx4; *P* = .049). At Day 28, up-regulation of immune response pathways (complement system, Toll-like receptor pathway) and metabolic regulation pathways (Hnf1a, Cry1) were observed in the cellular CC patch group, whereas no significant changes were detected in pathways related to myocardial contraction, energy metabolism, or remodeling.

When comparing the acellular CC patch and control groups at Day 7, there was a notable down regulation of a gene involved positive regulation of vascular associated SMC regulation (Nr4a3; *P* = .03) as well as fibroblast growth factor receptor binding (Fgfr14; *P* = .04). There were no enriched pathways at Day 28.

### Human cell integration to host myocardium

**Figure 5.**
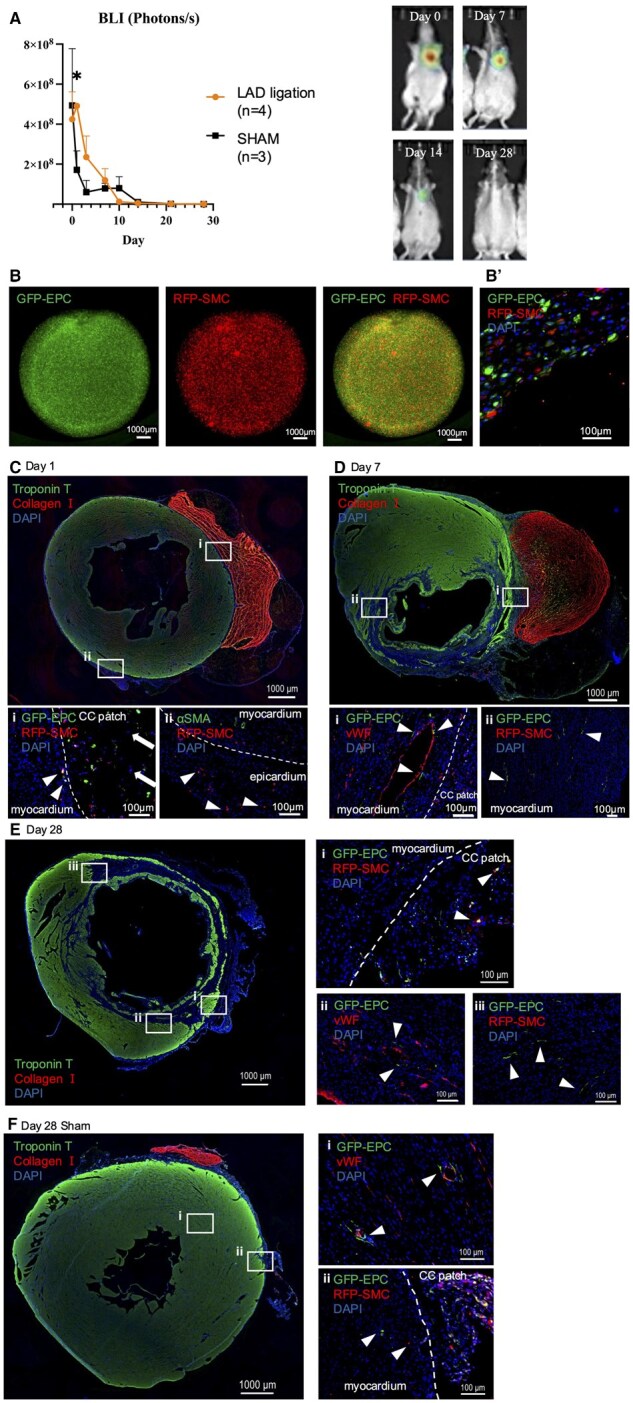
Cell fate tracking by bioluminescent imaging (BLI) and fluorescent cell labelling. (**A**) Longitudinal BLI imaging shows cell survival (mean ± SE) in injured (LAD Ligation, *n* = 4) and uninjured (Sham, *n* = 3) rats from Day 0 to Day 28. Representative bioluminescent images of rats with LAD Ligation representing the fading signal over time. (**B**) In vitro image of cellular CC patch shows EPCs labeled with GFP and SMCs labeled with RFP. (**B**’) IHC staining of the cross-section of patch.(**C-E**) The IHC staining of the heart after LAD ligation. Heart cross section shows troponin T (green) and collagen 1 (red) to identify myocardium and the collagen (**C**) IHC staining of the heart on post-transplant Day 1. (i) Myocardium and the cellular CC patch with GFP-labeled EPCs and RFP-labeled SMCs migrated into the myocardium (white arrowhead). White arrow shows infiltration of some GFP-/RFP-/DAPI+ host cells into the cellular CC patch. (ii) GFP-labeled EPCs were present in the epicardium away from transplanted patch. (**D**) IHC staining of the heart on post-transplant Day 7. (i) GFP-labeled EPCs have directly integrated with host vasculature as shown by the colocalization with vWF (red). (ii) GFP-labeled EPCs were present in the border zone away from transplanted patch. (**E**) IHC staining of the heart on post-transplant Day 28. (i) RFP-labeled SMCs and GFP-labeled EPCs were present in the cellular CC patch (white arrowhead). (ii) GFP-labeled EPCs were observed around vWF-positive vessels in the myocardium (white arrow). (iii) GFP-labeled EPCs were present in the border zone away from transplanted patch. (**F**) The IHC staining of the heart 28 days after implantation of the cellular CC patch without LAD ligation (Sham). (i) GFP-labeled EPCs have observed in the host vasculature showed with vWF. (ii) RFP-labeled SMCs and GFP-labeled EPCs were present in both myocardium and the cellular CC patch (white arrowhead). All IHC images include DAPI nuclear stain in blue. GFP, Green fluorescent protein; EPC, endothelial progenitor cell; RFP, red fluorescent protein; SMC, smooth muscle cell; vWF, von Willebrand Factor; αSMA, alpha smooth muscle actin. The white dashed line indicates the myocardial-patch putative border.

Retention of transplanted cells was evaluated by combined in vivo bioluminescent imaging (BLI) and ex vivo histological assessment. Longitudinal bioluminescent measurements showed a steady decline through post-MI Day 14; in the control group, cell retention at Day 14 was 1.7% of baseline. From Day 14 to Day 28, bioluminescent signal continued to decline but remained detectable above the background and localized on the heart at each time point up to Day 28. To understand the impact of the infarcted tissue environment on cell survival, three rats received a cellular CC patch transplantation without LAD ligation (Sham group). Compared to the injured rats, there was a more rapid initial decline in cell survival until post-transplantation Day 3, followed by stabilization through Day 10. At Day 1, the LAD ligation group had higher cell survival than the sham operation group, whereas later time points show no difference in cell survival between groups (Fig. 5A).

Ex vivo assessment of GFP and RFP fluorescence reflects the results obtained by BLI measurements regarding cell retention and provided further spatial resolution. Immunohistochemical evaluation of a whole cellular CC patch demonstrates homogenously mixed GFP-labeled EPCs and RFP-labeled SMCs before transplantation (Fig. 5B and B’). At Day 1 post-transplantation, the bulk of labelled EPCs and SMCs were retained in the cellular CC patch (Fig. 5C and 5Ci). Attachment to and migration into the myocardium was observable in a small number of GFP-labeled EPCs and RFP-labeled SMCs at the interface of the patch and myocardium. Additionally, infiltration of some GFP-/RFP-/DAPI+ host cells into the collagen patch was seen. A group of RFP+ SMCs migrated out of the patch along the epicardial surface (Fig. 5Cii)

At Day 7, GFP^−^/RFP^−^ nuclei derived from host cells were seen in the cellular CC patch (Fig. 5D and 5Di). GFP-labeled EPCs directly integrated with host vasculature, in both large existing host vessels, and smaller potentially new microvasculature, as shown by the colocalization with vWF (Fig. 5Di). GFP-labeled EPCs were detectable throughout the infarct myocardium, most notably around the border zone of the infarct area, including in the septal portion of the myocardium (Fig. 5Dii)

At Day 28, the CC patch was no longer identifiable on the heart surface on most samples (Fig. 5E). Transplanted RFP-­labeled SMCs and GFP-labeled EPCs were present in both myocardium and the collagen patch (Fig. 5Ei). GFP-labeled EPCs were observed around vWF-positive vessels in the myocardium (Fig. 5Eii). GFP-labeled EPCs were again detected in the border zone of the infarct area (Fig. 5Eiii).

Transplanted cell localization on the sham hearts was also evaluated at Day 28 to consider the impact of the infarct microenvironment on cell migration patterns. Human cells were found in the small remaining portion of the CC patch, similar to the injured hearts (Fig. 5F and 5Fi). GFP-labeled EPCs were found deep in the myocardial tissue in proximity to vWF-positive vessels (Fig. 5Fii).

## Discussion

The primary motivation for this study was to develop a novel therapeutic for microvascular malperfusion in ischemic heart disease. We’ve shown that transplanting a cellular compressed collagen patch with EPCs and SMCs can effectively treat microvascular malperfusion and progressive cardiac decline after ischemic injury while promoting mature angiogenesis in the host myocardium. This platform offers a promising method to deliver therapeutic cells to the post-infarction heart and explore the underlying biology of tissue-engineered constructs.

Our adaptation of the compressed collagen patch as a tissue-engineered construct overcomes significant limitations in treating cardiac injury. The patch, made from biocompatible collagen in under an hour, allows for transplantation within hours of an MI. We’ve shown that human peripheral blood (yielding EPCs) and bone marrow (yielding MSCs with an SMC phenotype) can be noninvasive cell sources for autologous transplantation in patients planning elective revascularization.^18^ The compressed collagen patch is mechanically robust, flexible, and easily sutured, which enhances ease of use. Achieving tissue-like strength and robust handleability minimizes the risk of tears or cell damage during transplantation. Beyond this study, the anisotropic structure increases hydraulic resistance, making the material suitable for diverse tissue engineering applications like skin, bone, cornea, bladder, cartilage, and blood vessel regeneration.^23^^,^^28^^,^^33^

Functional data indicates that the cellular CC patch therapy significantly improves cardiac function compared to the control and acellular CC patch groups. The cellular CC patch group showed a higher final LVEF, demonstrating consistent improvement over time, while the other two groups showed no improvement. This stagnation in the control and acellular groups implies that the recovery observed in the cellular CC patch group is due to the therapeutic performance of the angiogenic cells. Although no structural differences were found between groups, the ESPVR, representing heart contractility, was elevated in the cellular CC patch group, showing improved cardiac muscle performance. Notably, neither CC patch group caused diastolic dysfunction, which is a critical consideration for topical epicardial therapy. The functional improvement likely results from the proangiogenic effects and positive remodeling triggered by the cellular CC patch.

This theory is supported by histologic evidence of reduced fibrosis and IHC evidence of enhanced microangiogenesis in the cellular CC patch border zone. While some auto-angiogenesis is expected in ischemic tissue, the cellular CC patch directly contributed human-origin vessels through cell engraftment. Additionally, the higher degree of auto-angiogenesis observed in the cellular CC patch group suggests a beneficial paracrine effect from the transplanted cells. Bioluminescent imaging and fluorescent cell labeling further demonstrate this paracrine signaling effect on vascular proliferation.^68^ Injured hearts showed increased cell retention after transplantation, indicating reciprocal interaction between host and transplanted cells. The transplanted cells exert a cardioprotective effect on the host, while the ischemic myocardium supports EPC and SMC-like MSC survival through pro-survival and pro-migration paracrine signaling.^69–71^ Non-infarcted hearts without these paracrine signals displayed reduced cell migration, integration, and survival.

Our transcriptomic analysis revealed that by post-MI Day 7, the cellular CC patch therapy trended toward a pro-angiogenic and pro-survival phase, evolving into distinct pro-regenerative transcriptional pathways by Day 28. On Day 7, findings indicate that rather than the harm typically caused by MI, the cellular CC patch promotes angiogenesis and cardiomyocyte survival. Although the patch performed better at Day 7, the control group was likely still in the proliferative phase of recovery, including some degree of auto-angiogenesis.^72^ By Day 28, however, the gap in therapeutic performance became more evident as the cellular CC patch group displayed robust HIF-independent angiogenesis, upregulating Apold1,^73^ Angptl4,^55^^,^^58^ RhoB,^57^ and CIB1.^56^ By post-MI Day 21, the heart is typically in a post-maturation phase, meaning native recovery is complete,^72^ and thus continuous angiogenic pathways can be directly attributed to the cellular CC patch. This data aligns with the functional benefits and histological evidence observed at Day 28.

Our research highlights the divergent migration patterns of EPC and SMC-like MSC populations. A small subset of SMC-like MSCs quickly mobilized from the patch, migrating along the epicardial surface within 24 hours of transplantation. However, most remained in the cellular CC patch up to Day 7, emphasizing the importance of understanding their behavior for improving engraftment and therapeutic potential. EPCs showed limited migration initially, but by Day 7, few remained in the patch. They were distributed throughout the infarct scar, often near host vascular structures and accumulating at the infarct border zone. This finding reinforces that SMC-like MSCs primarily benefit ischemic myocardium through paracrine signaling,^17^ while EPCs offer both paracrine effects and direct integration.^74^

Our results highlight the value of studies focusing on increasing MSC migration to the myocardium via strategies such as cell priming,^75^ gene editing,^76^ or chemokine incorporation.^77^ In normal physiology, both MSCs and EPCs are mobilized from peripheral tissues into systemic circulation to target injuries.^17^^,^^78^ However, we observed that each cell type exhibits distinct migration timelines and target specificity: MSCs migrate quickly to the epicardial surface, while EPCs home more slowly to the infarct border zone. Leveraging the unique characteristics of each cell type can further improve patch efficacy.

Our results do not show strong evidence of improved functional recovery in the acellular CC patch group, despite Serpooshan et al. previously reporting cardioprotective effects of acellular compressed collagen patches in a mouse myocardial infarction model.^79^ In our study, the acellular CC patch group exhibited increased fibrosis, unattenuated LV remodeling, and no improvement in ejection fraction compared to controls. While this group did show enhanced capillary density and ESPVR, the therapeutic potential remains unconvincing. This discrepancy may be due to methodological differences, such as species and immunocompetence, but also indicates that the primary therapeutic impact in our cellular CC patch comes from the transplanted EPCs and SMCs. Our findings suggest that combining cell therapy with a hydrogel patch is superior to using the hydrogel alone, despite the logistical challenges of cell isolation and expansion.

This study offers a promising path toward realizing the therapeutic potential of tissue-engineered constructs for heart disease treatment, revealing areas for optimization and understanding of underlying biology. Future research should focus on determining the optimal mechanical and physicochemical properties of the cellular CC patch to maximize cell survival and cardioprotective effects. While this study used a homogenous mix of EPCs and SMCs, exploring different cell ratios or priming strategies could enhance outcomes. Given the importance of cytokine signaling, directly incorporating angiogenic or other molecules might improve patch function.

Our study also has several limitations that may deserve specific scrutiny in future work. The xenotransplantation of human cells into immunodeficient nude rats offers the best small-animal model for studying human cell behavior. However, immune cell activity post-infarction is crucial to the progression of ischemic cardiomyopathy and may differ significantly between humans and rats, particularly in innate immune responses to foreign cells.^80^ This limitation could be addressed by future studies in humanized rodents. Another question is the long-term durability of cardiac functional recovery after cell patch transplantation. By Day 28, BLI and histology revealed that most transplanted cells did not survive, yet functional benefits persisted with improved cardiac function, increased mature angiogenesis, and durable angiogenic pathway upregulation. Longer-term studies will clarify whether these benefits are sustained or fundamentally limited by the short-term survival of transplanted cells. Additionally, scaling up from rodent-sized to human-sized constructs is a significant challenge that requires attention before advancement to a large animal model.

In conclusion, by inducing angiogenesis and recovering myocardial function, we have demonstrated the viability of a rapidly manufacturable EPC-SMC-based compressed collagen patch as an interventional therapy for ischemic heart failure. Thus, the cellular CC patch offers a translatable platform to address a currently untreated aspect of ischemic cardiac injury.

## Supplementary Material

szaf035_Supplementary_Data

## Data Availability

All data are available in the main text or the Supplementary material.
