# Supplementary material for: Rapid manufacturing of angiogenic cellular collagen patches for ischemic cardiomyopathy
Source: Stem Cells Transl Med. 2025 Sep 19;14(9):szaf035. doi: 10.1093/stcltm/szaf035 (PMC12449208; doi:10.1093/stcltm/szaf035)
Supplement: szaf035_Supplementary_Data [file szaf035_supplementary_data.zip › Supplementary Information collagen 20250623YK.docx]

**Supplementary Information**

**Rapid manufacturing of angiogenic cellular collagen patches for ischemic cardiomyopathy**

**Authors:**

Eric Pfrender, BS†^1, 2^, [ericpfrender2020@u.northwestern.edu](mailto:ericpfrender2020@u.northwestern.edu)

+1-650-723-2300

870 Quarry Road, Palo Alto, CA 94304

Sungwoo Kim, PhD†^3^, [kim4@stanford.edu](mailto:kim4@stanford.edu)

+1-650-723-2300

300 Pasteur Drive, Stanford, CA 94305

John A. Farag, MD†^1, 2, 4^, [faragja@stanford.edu](mailto:faragja@stanford.edu)

+1-415-818-2993

870 Quarry Road, Palo Alto, CA 94304

Shin Yajima, MD, PhD^1, 2^, [syajima@stanford.edu](mailto:syajima@stanford.edu)

+81-90-3748-3134

870 Quarry Road, Palo Alto, CA 94304

Yujiro Kawai MD, PhD^1, 2^, [ykawai@stanford.edu](mailto:ykawai@stanford.edu)

+1-650-223-4696

870 Quarry Road, Palo Alto, CA 94304

Koji Kawago, MD, PhD^1, 2^, [kkawago@stanford.edu](mailto:kkawago@stanford.edu)

+1-650-283-0855

870 Quarry Road, Palo Alto, CA 94304

Umayr Syed, BS^1, 2^, [Umayrs5@gmail.com](mailto:Umayrs5@gmail.com)

+1-650-723-2300

870 Quarry Road, Palo Alto, CA 94304

Gentaro Ikeda MD, PhD^2, 5^, [ikedagen@stanford.edu](mailto:ikedagen@stanford.edu)

+1-650-272-1807

870 Quarry Road, Palo Alto, CA 94304

Tsuyoshi Ueyama, MS^5^, [ueyama.tsuyoshi@gmail.com](mailto:ueyama.tsuyoshi@gmail.com)

+1-650-723-2300

870 Quarry Road, Palo Alto, CA 94304

Hiroyuki Takashima, PhD^5^, [hirotakashima@pop.med.hokudai.ac.jp](mailto:hirotakashima@pop.med.hokudai.ac.jp)

+1-650-723-2300

870 Quarry Road, Palo Alto, CA 94304

Alex Dalal MD^1, 2^ , [alexdalal@gmail.com](mailto:alexdalal@gmail.com)

+1-650-723-2300

870 Quarry Road, Palo Alto, CA 94304

Yuanjia Zhu, MD, PhD^1, 2^, [yuanjiaz@stanford.edu](mailto:yuanjiaz@stanford.edu)

+1-650-723-2300

870 Quarry Road, Palo Alto, CA 94304

Kenzo Ichimura MD, PhD^2, 6^, [kennzo@stanford.edu](mailto:kennzo@stanford.edu)

+1-650-723-2300

300 Pasteur Drive, Stanford, CA 94305

Yu Liu, PhD^2, 5^, [shuaiyu@stanford.edu](mailto:shuaiyu@stanford.edu)

+1-650-723-2300

300 Pasteur Drive, Stanford, CA 94305

Seyedsina Moeinzadeh, PhD^3^, [sinam@stanford.edu](mailto:sinam@stanford.edu)

+1-650-723-2300

300 Pasteur Drive, Stanford, CA 94305

Jayme Koltsov, PhD^3^,  [jcbk@stanford.edu](mailto:jcbk@stanford.edu)

+1-650-723-2300

300 Pasteur Drive, Stanford, CA 94305

Joseph C Wu, MD, PhD^2, 5, 7^, [joewu@stanford.edu](mailto:joewu@stanford.edu)

+1-650-723-2300

300 Pasteur Drive, Stanford, CA 94305

Y Joseph Woo, MD^1, 2, 8^, [joswoo@stanford.edu](mailto:joswoo@stanford.edu)

+1-650-725-3828

870 Quarry Road, Palo Alto, CA 94304

Phillip C. Yang, MD^2, 5^, [phillip@stanford.edu](mailto:phillip@stanford.edu)

+1-650-723-2300

870 Quarry Road, Palo Alto, CA 94304

Y Peter Yang, PhD*^3, 8, 9^, [ypyang@stanford.edu](mailto:ypyang@stanford.edu)

+1-650-723-2300

300 Pasteur Drive, Stanford, CA 94305

Yasuhiro Shudo MD, PhD*^1, 2^ [yshudo@stanford.edu](mailto:yshudo@stanford.edu)

+1-650-304-6091

870 Quarry Road, Palo Alto, CA 94304

**Affiliations:**

^1^ Department of Cardiothoracic Surgery, Stanford University School of Medicine; Stanford, CA, USA 94305

^2^ Stanford Cardiovascular Institute, Stanford University School of Medicine; Stanford, CA, USA 94305

^3^ Department of Orthopedic Surgery, Stanford University School of Medicine; Stanford, CA, USA 94305

^4^ Department of Surgery, Stanford University School of Medicine; Stanford, CA, USA 94305

^5^ Department of Medicine, Division of Cardiovascular Medicine, Stanford University School of Medicine; Stanford, CA, USA 94305

^6^ Department of Medicine, Division of Pulmonary, Allergy, and Critical Care Medicine, Stanford University School of Medicine; Stanford, CA, USA 94305

^7^ Institute of Stem Cell Biology and Regenerative Medicine, Stanford University School of Medicine; Stanford, CA, USA 94305

^8^ Department of Bioengineering, Stanford University School of Medicine; Stanford, CA, USA 94305

^9^ Department of Materials Science and Engineering, Stanford University School of Medicine, Stanford, CA, USA 94305

**Corresponding Authors:**
Yasuhiro Shudo, MD, PhD; e-mail: [yshudo@stanford.edu](mailto:yshudo@stanford.edu)
Peter Yang, PhD; e-mail: [ypyang@stanford.edu](mailto:ypyang@stanford.edu)
