# Supplementary figures and images for: Rapid manufacturing of angiogenic cellular collagen patches for ischemic cardiomyopathy
Source: Stem Cells Transl Med. 2025 Sep 19;14(9):szaf035. doi: 10.1093/stcltm/szaf035 (PMC12449208; doi:10.1093/stcltm/szaf035)

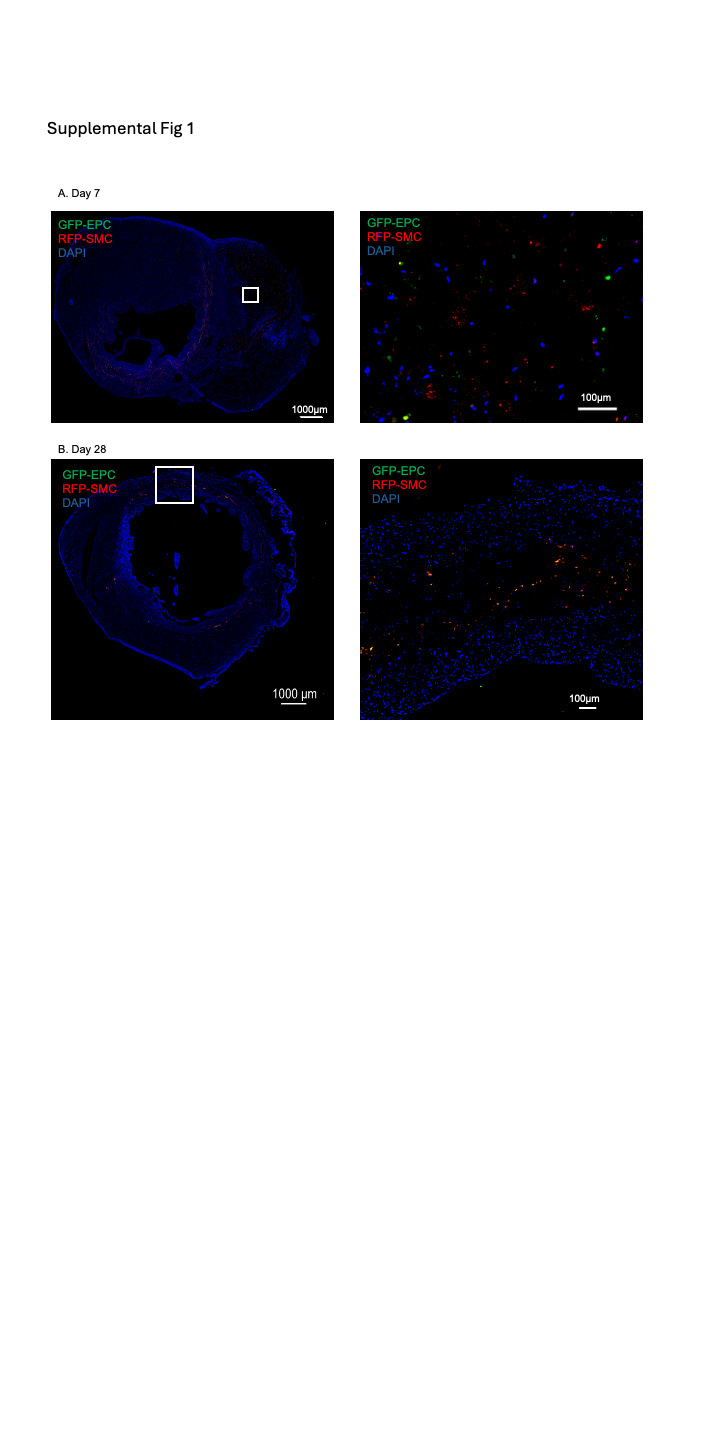

Supplement: szaf035_Supplementary_Data [file szaf035_supplementary_data.zip › SI.tiff]
